# Relative humidity in intermittently conditioned energy-efficient homes: a preliminary hygrothermal assessment of indoor condensation and mould risk

**DOI:** 10.14324/111.444/ucloe.3660

**Published:** 2026-11-03

**Authors:** Liqun Guan, Mark Dewsbury, Hartwig Kuenzel, Louise Wallis

**Affiliations:** 1 University of Tasmania, Launceston, Australia; 2 Fraunhofer Institute for Building Physics, Munich, Germany

**Keywords:** energy-efficient house, uncontrolled relative humidity, intermittent conditioning, hygrothermal performance, indoor mould growth risk, clay masonry veneer, compressed fibre cement sheet, timber-framed wall, indoor environment quality

## Abstract

Rising global temperatures and increasing energy demand have led to more stringent energy efficiency requirements in buildings. While these measures reduce energy use, they may unintentionally increase the risk of indoor and interstitial moisture accumulation and mould growth. Highly insulated and airtight dwellings, combined with occupant-controlled ventilation and intermittent space conditioning without relative humidity regulation, can create environments conducive to mould, threatening indoor air quality, occupant health and building durability. In Australia, the 2022 National Construction Code mandates a minimum 7-star building envelope focused energy rating for new residential dwellings. Unlike international standards, Australian regulations include intermittent conditioning without specifying indoor relative humidity limits, potentially increasing moisture-related risks. This study examines the effects of intermittently conditioned indoor climate on interstitial moisture accumulation and mould growth in two of the most common low-rise timber-framed wall systems: clay masonry veneer and compressed fibre cement sheet cladding within temperate Australian climate zones. Ten-year transient hygrothermal simulations were conducted to quantify moisture and mould risks. Results show, without relative humidity control, indoor conditions frequently exceed the internationally recommended 70% relative humidity threshold, significantly increasing interstitial mould risk, particularly in bedrooms and open-plan living space. Comparisons with continuous conditioning, per the American Society of Heating, Refrigerating and Air-Conditioning Engineers Standard 160, indicate lower risks under continuous conditions. Mitigation strategies, including ventilated cavities and internal vapour control layer, were effective under continuous conditioning but exhibited inconsistent performance under conditions of unregulated relative humidity in intermittent conditioning. The findings reveal the limitations of continuous conditioning assumptions for moisture control design and underscore the problem of underestimating moisture risks in buildings.

## Introduction

### Background

As a response to global climate change and the energy crisis, energy-efficient building design is one of the central strategies for achieving carbon-neutral goals, with a growing emphasis on airtightness and weather-resilient construction [1–4]. This shift is also reflected in the Australia National Construction Code (NCC) 2022, which mandates a minimum 7-star building envelope focused energy rating for new residential buildings. Notably, research has found that, while reducing energy losses from indoor heating and cooling, this approach can also lead to unintended consequences, particularly an increased risk of indoor and interstitial condensation and mould growth, thereby affecting indoor environmental quality and air quality [5–8]. Indoor air quality (IAQ), a key determinant of occupant health and comfort, is closely linked to indoor relative humidity (RH) [9–11]. Elevated RH levels create favourable conditions for microbial proliferation, most notably mould, which undermines both occupant health and building durability [12–14]. Interstitial mould or fungal growth within concealed assemblies, such as wall cavities, further threatens structural integrity and often remains undetected until significant damage occurs. The World Health Organization (WHO) recognises dampness and mould as critical contributors to respiratory illness and allergies, underscoring their public health significance [15]. Research demonstrates that mould germination can occur at RH levels above 70% [8–10], whereas maintaining indoor RH below 60% is generally advised to minimise the risk of growth.

Existing hygrothermal assessment methods and standards commonly rely on simplified indoor climate assumptions [16], typically based on continuously conditioned temperature and/or RH interior environments. However, intermittent temperature and RH conditioning is more representative of residential building operation generally and is explicitly reflected in the Energy Efficiency clauses of the NCC through its prescribed residential conditioning regimes. Under such conditions, indoor environments may experience greater temporal variability in temperature and humidity, potentially leading to different interstitial moisture responses compared to continuously conditioned cases. Despite its practical relevance, the influence of intermittent conditioning on moisture accumulation and mould growth risk remains insufficiently investigated [17,18].

### Intermittent conditioning regimes

The challenge of balancing energy efficiency and moisture control is particularly pronounced in the Australian context. Unlike standards in many Northern Hemisphere countries, such as the United States, Japan, North China and much of Europe (including Germany, the United Kingdom and the Netherlands), where continuous indoor conditioning is common, Australian regulations require intermittent conditioning of habitable rooms without specifying numerical requirements for indoor RH.

From a building physics perspective, the key distinction is not simply whether the air conditioner is on or off, but how indoor temperature and moisture conditions evolve over time and interact with the building envelope. Under continuous conditioning: (1) indoor temperature and RH are maintained within a relatively narrow range; (2) moisture loads are more consistently controlled through cooling, heating and/or dehumidification; (3) building envelope materials experience smaller fluctuations in temperature and moisture content and; (4) drying potential is generally more predictable and stable. As a result, moisture accumulation within building assemblies is less likely to occur, and hygrothermal simulations often show lower mould growth risk.

Under intermittent conditioning: (1) conditioning systems operate only during certain periods (e.g., when occupants are present or when temperatures become uncomfortable); (2) indoor temperature and RH can fluctuate significantly between conditioned and unconditioned periods; (3) during conditioning-off periods, indoor RH may increase, especially in warm-humid climates and; (4) building materials can absorb moisture during high-humidity periods and may not fully dry before the next moisture exposure event. Consequently, the building envelope experiences cyclic wetting and drying processes that can lead to higher moisture accumulation and increased mould growth potential.

While this intermittent conditioning strategy reduces energy consumption [18], it can affect moisture-related risks, particularly in airtight, highly insulated dwellings with occupant-controlled ventilation [8,19–21]. Additional factors such as thermal bridging, ventilation practices and inappropriate insulation could further exacerbate these vulnerabilities [22–24]. This distinction is important because moisture-related risks are highly sensitive to both the magnitude and duration of elevated RH conditions. A wall assembly that performs satisfactorily under continuous conditioning may exhibit substantially different moisture behaviour under intermittent conditioning due to:

- higher peak RH levels
- longer periods above critical moisture thresholds
- reduced drying potential, and
- increased moisture storage within hygroscopic indoor materials.

Therefore, assuming continuous conditioning may underestimate moisture and mould risks in residential buildings where intermittent conditioning operation is common. Accurately representing intermittent conditioning is essential for realistic hygrothermal assessment, moisture-risk prediction and the development of robust building envelope design guidance.

Internationally, a range of methods has been employed to investigate building environmental RH and mould risk, including predictive modelling validated by laboratory testing [25,26], field-based sampling to detect hidden mould [27,28] and computer-based hygrothermal simulations of moisture transport and accumulation in building envelopes [29–31]. Among these methods, transient hygrothermal simulations are particularly effective in providing a time-efficient and reliable approach to approximating the long-term impacts of RH variation under representative operating conditions.

Whilst previous research [32] evaluated the baseline hygrothermal risks of fully intermittent conditioned scenarios, the isolated impact of indoor RH under active thermal control remains unquantified. Extending that foundational work, this study decouples these parameters by regulating indoor temperature while leaving RH unconstrained. This isolates the governing influence of indoor moisture dynamics, clarifying the necessity of dedicated humidity management for durable, healthy built environments.

By examining the interplay between intermittent conditioning, RH management and mould proliferation, this research yields insights that are highly applicable to Australia as well as other countries that are moving away from continuous conditioning towards intermittent building conditioning. Notably, at the 28th Conference of Parties (COP28) in 2023, representatives from more than 63 countries, including the United States, Canada and leading members of the European Union, committed to reducing emissions associated with cooling by shifting away from continuous, energy-intensive cooling systems through the adoption of passive cooling strategies and enhanced efficiency standards [33–35]. In response, building codes in several regions actively encourage intermittent conditioning, alongside the implementation of minimum energy performance requirements for active systems.

### The goal of this study

The goal of this study is to investigate how different conditioning operations influence indoor environmental quality. Specifically, it aims to compare continuous conditioning with both temperature and RH control against intermittent conditioning with no control of RH, and to assess how these approaches affect interstitial condensation and mould growth risks across studied climates.

To achieve this, WUFI (Wärme und Feuchte instationär; transient heat and moisture transport) version Pro 6.0 (Fraunhofer Institute for Building Physics, Germany) transient hygrothermal and bio-hygrothermal simulations are applied to examine the impact of uncontrolled indoor RH in two of Australia’s most common low-rise wall systems: timber-framed clay masonry veneer and compressed fibre cement sheet (CFCS) cladding. Building on the Australian and international context of intermittent conditioning, the study focuses on three temperate climate zones (CZs): Nationwide House Energy Rating Scheme (NatHERS) CZ21 represents a medium-density urban environment, CZ64 is a cool temperate coastal climate zone and CZ66 is an inland climate zone with the highest RH of the three, as detailed later in this paper. To evaluate the external wall performance, two indoor conditioning scenarios are compared: (1) continuous control of indoor temperature and RH in accordance with the American Society of Heating, Refrigerating and Air-Conditioning Engineers (ASHRAE) 160 and the Australian Institute of Refrigeration, Air Conditioning and Heating (AIRAH) Design Application (DA) 07 thresholds, as studied in previous research [36,37], and (2) controlled indoor temperature with uncontrolled RH in intermittent conditioning, which is the main focus of this study. Furthermore, mitigation strategies, including ventilated cavities and interior vapour control layer combinations, are also assessed.

## Settings and climate zones

### Settings

To more accurately reflect an uncontrolled RH within an intermittent air-conditioning scenario of an Australian energy-efficient home, the researchers conducted a building energy rating simulation using AccuRate. AccuRate is a simulation tool developed by the Commonwealth Scientific and Industrial Research Organisation (CSIRO) for the Australian NatHERS. AccuRate is used to calculate the indoor environmental conditions of a typical 7-star-rated detached house in south-eastern Australia. The floor plan, shown in Fig. 1, is a four-bedroom home with an interior volume of 366 m^3^ and a floor area of 152.5 m^2^. This plan represents a typical low-rise timber-framed house design in south-eastern Australia, consistent with previous research [36,38].

**Figure 1:**
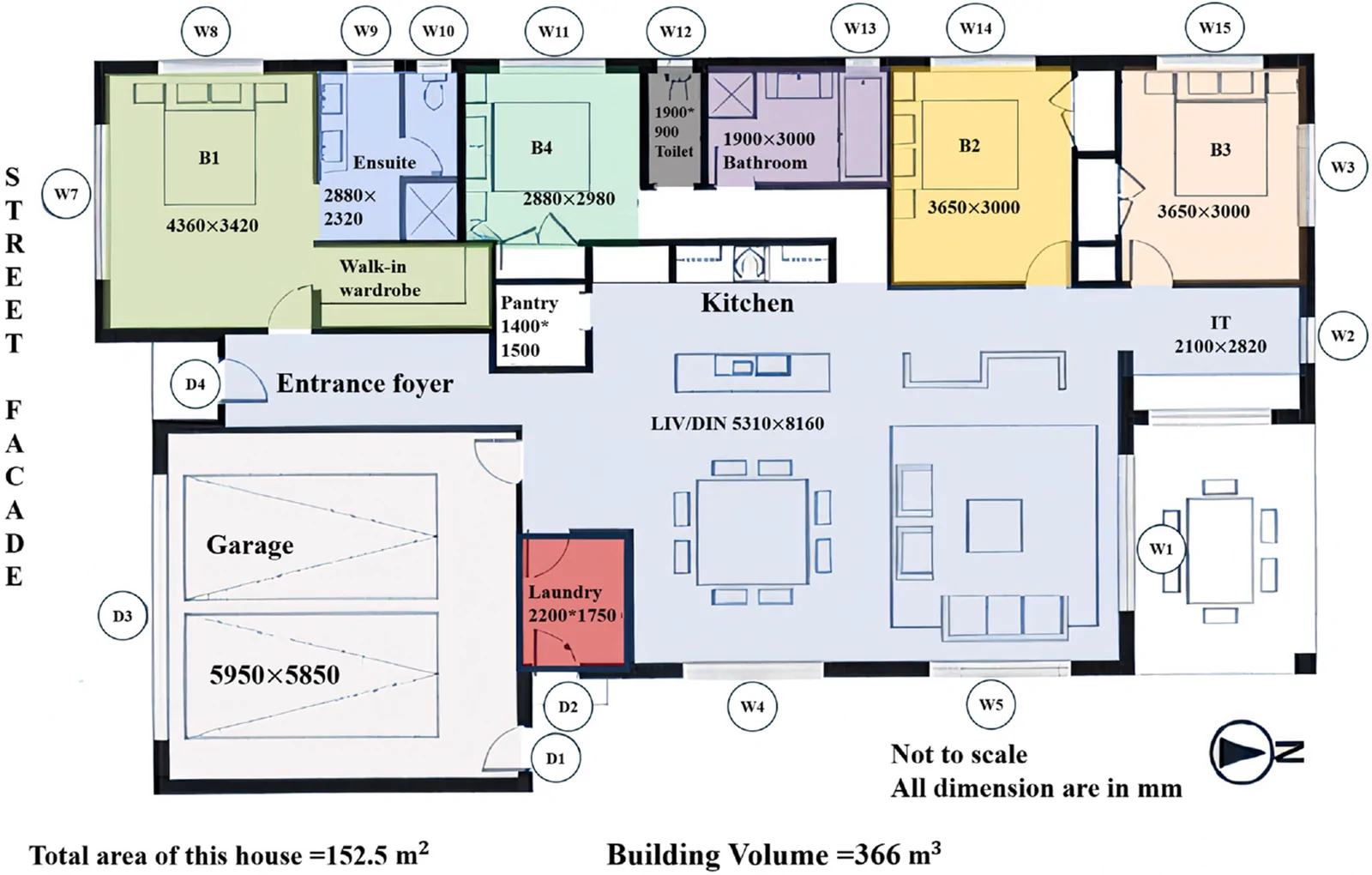
Floor plan of the studied house (not to scale).

The timber-framed clay masonry veneer (or brick veneer) and CFCS-clad external wall systems represented 54.2% and 20.3% of new dwellings, respectively, as of 2024 [39]; this study examines the two climatic regions experiencing the highest rates of residential development and their associated capital city in south-eastern Australia (see Table 1). Their widespread adoption makes them critical to understanding the moisture performance of typical Australian building envelopes under the updated energy efficiency requirement and local conditioning practice [39,40]. The primary focus is on analysing moisture behaviour and interstitial mould growth risk associated with these selected external wall systems and further testing the effectiveness of mitigations that are currently suggested by the Australian Building Codes Board and design professionals (architects, engineers, building designers, building physicists).

**Table 1.** NatHERS climate zones and distribution of new dwellings

NatHERS climate	NCC climate	Location of weather station	Percentage of new houses built by 2024
**21**	**6**	**Melbourne**	**6%**
22	6	East Sale	12%
27	4 and 6	Mildura	9%
60	6	Tullamarine	12%
61	4 and 6	Mt Gambier	2%
63	6	Warrnambool	4%
**64 (+62)**	**6**	**Cape Otway (+Moorabbin)**	**25%**
**66**	**4 and 6**	**Ballarat**	**30%**

Shaded cells with bold text highlight those selections included in this study.

### Two Australian climate zones: NCC and NatHERS

In studies of energy-efficient housing design and assessment in Australia, two primary climate classification systems are commonly used. The NCC CZs divide Australia into eight broad regions, to guide regulatory requirements for building design, construction practices and material choices [41]. In contrast, the NatHERS framework defines 69 more detailed CZs allocated by postcode [42], which are applied specifically in thermal performance modelling of residential buildings. While both systems seek to capture climatic variability, the NCC zones are primarily used for compliance with construction regulations, whereas the NatHERS zones provide finer-grained resolution for building energy rating assessments. This study references NCC CZs to align with regulatory requirements in modelling but also incorporates the climatic diversity, particularly the variation in RH and temperature, captured by NatHERS zones to ensure a more robust evaluation of building performance. The two CZ maps are shown in Fig. 2 [42]. The red dashed line highlights the locations of this research in south-eastern Australia.

**Figure 2:**
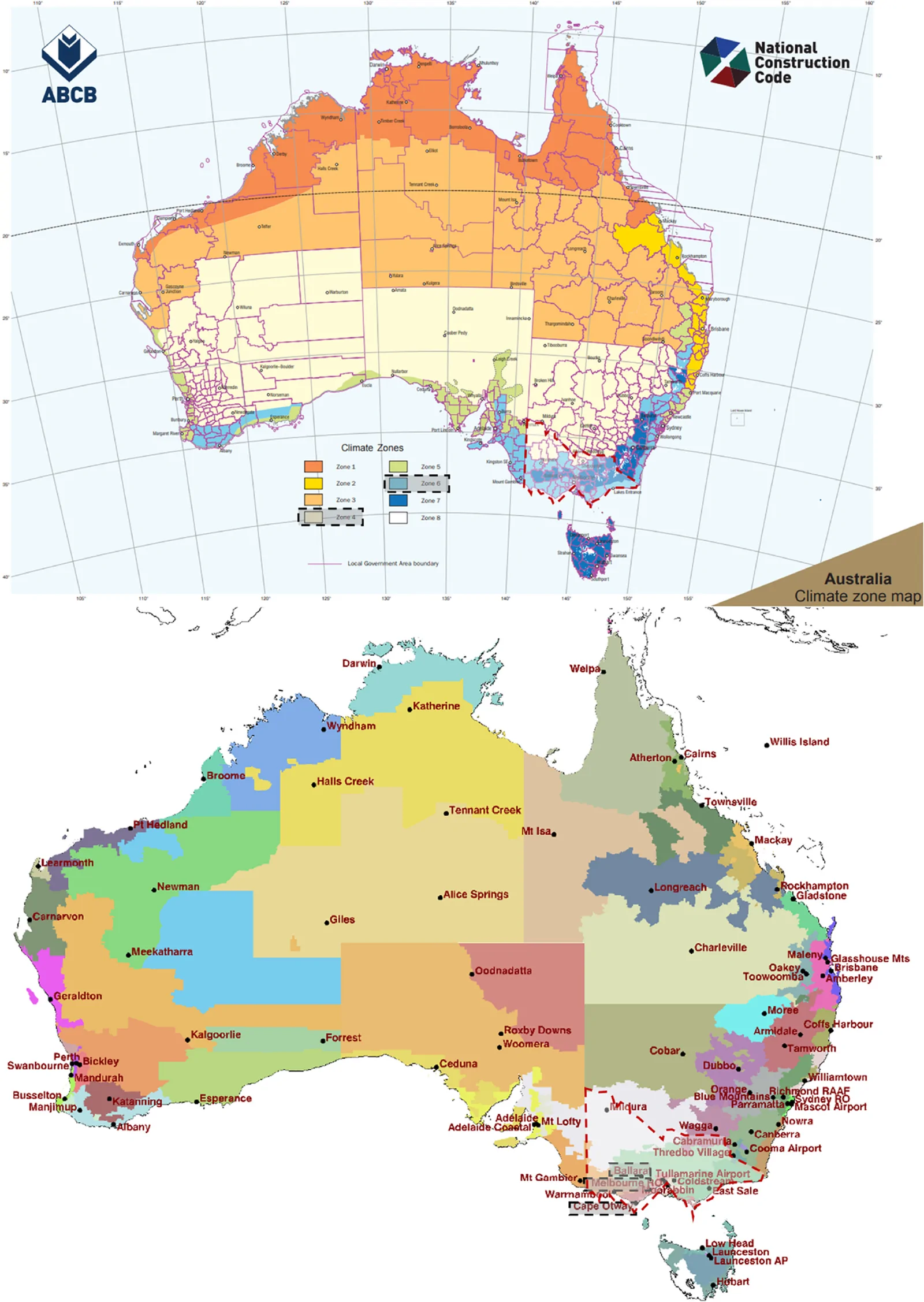
Comparison of NCC climate zone (top) and NatHERS climate zone (bottom). Source: NCC climate zone: https://ncc.abcb.gov.au/abcb-climate-map; NatHERS climate zone: https://www.homeenergyrating.gov.au/climate-zone-map

### The macroclimate

In this study, hourly climatic data were utilised as the primary input for the hygrothermal simulations to accurately capture transient environmental conditions. However, due to the high volume of hourly data across multiple CZs, Fig. 3 presents only aggregated climatic indicators. This provides a concise overview to help an international readership easily understand the general outdoor climatic context (e.g., thermal and moisture profiles) that underpins the hygrothermal assessment. This figure illustrates the monthly climate characteristics, specifically RH and average temperature for three distinct CZs: CZ21, CZ64 and CZ66. The graphs illustrate the monthly RH, with an interquartile range (IQR) of 1.5, and average temperature for each CZ. They provide crucial insights into annual climatic fluctuations, which are essential for predicting a building’s hygrothermal performance.

**Figure 3:**
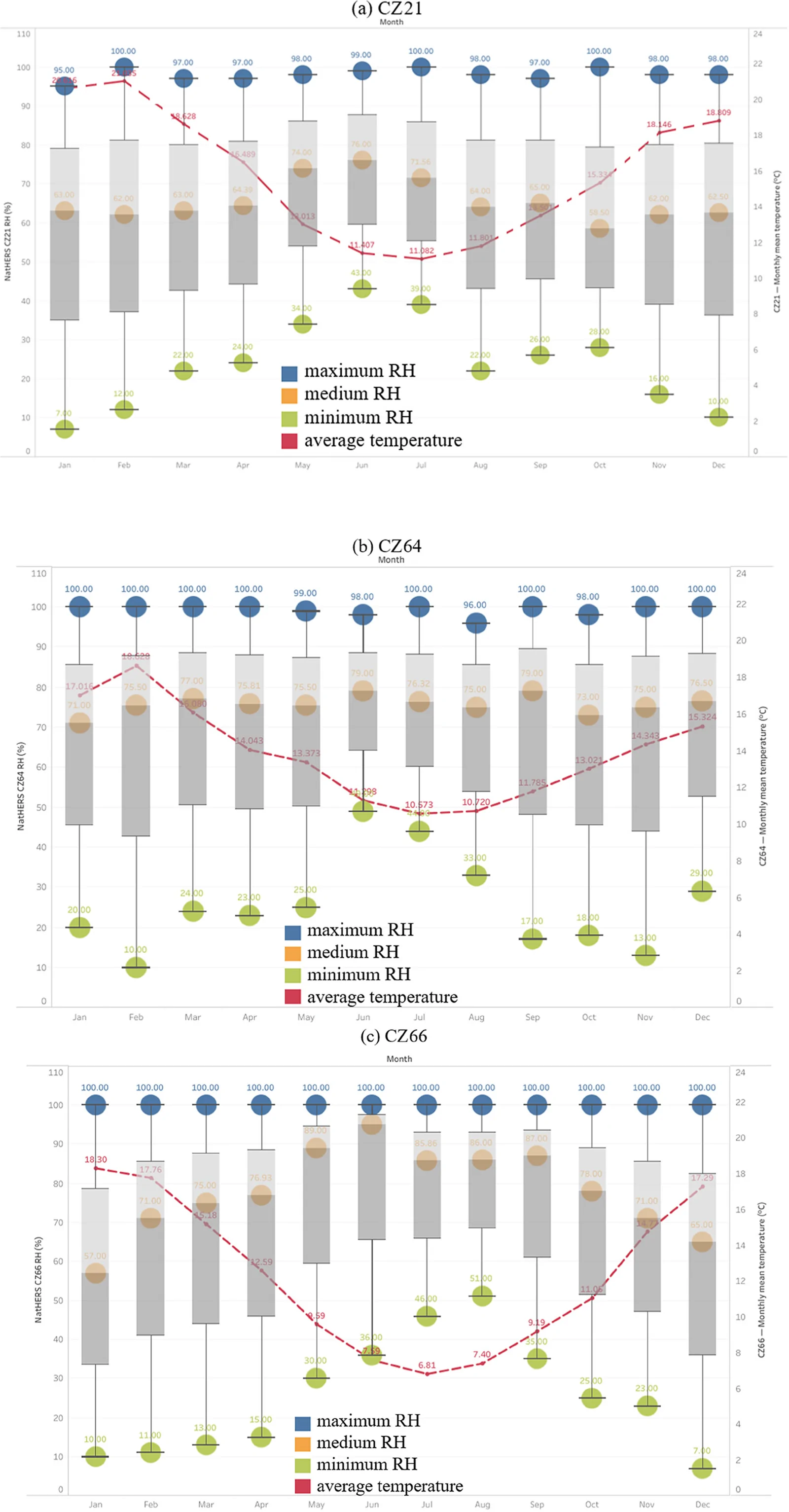
Monthly climate characteristics for NatHERS CZ21, CZ64 and CZ66.

#### Relative humidity (RH)

The box plots show the monthly maximum, median and minimum RH (in %).

- All three zones exhibit a consistently high maximum RH, frequently reaching 100% throughout the year. This suggests that the air is often saturated or near saturation, which is a key indicator of potential condensation and mould growth risks on surfaces or within building envelope layers, particularly during cooler periods.
- The median RH (orange dots in grey bar) generally remains high, hovering around 60%–80%, indicating a predominantly humid climate.
- The spread between maximum and minimum RH is significant, with minimums (green dots) occasionally dropping below 20% in some months (e.g., CZ66 in May and June), suggesting periods of relatively dry air that could affect the drying potential of materials.

#### Monthly average temperature

The red dashed line represents the monthly average temperature (in °C).

- All zones follow a typical annual cycle, peaking in the summer months (January/February) and reaching their minimums in winter (June to August).
- CZ21 shows the highest average temperatures, peaking above 20 °C and only dropping slightly below 10 °C in winter.
- CZ64 and CZ66 display lower overall average temperatures, particularly CZ66, which has a minimum average temperature of around 6–8 °C in mid-winter. The lower winter temperatures, combined with the persistently high RH, significantly increase the risk of interstitial and surface condensation, as the temperature of building elements is more likely to drop below the dew point.

#### Hygrothermal and bio-hygrothermal assessment

The data in Fig. 3 are fundamental to the hygrothermal assessment, as they define the environmental loads that the building envelope must withstand.

- Moisture management: the persistently high RH requires a building design that prioritises vapour and moisture control. Materials and assemblies must be selected for their ability to withstand high moisture content without degradation (e.g., rot, corrosion) and to allow for adequate drying capacity to manage trapped moisture.
- Condensation risk: the combination of low winter temperatures and high RH (especially the 100% maximums) highlights the critical need to assess the risk of both surface condensation (mould risk) and interstitial moisture (material damage, insulation failure). A detailed WUFI or similar simulation, informed by these data, is necessary to determine the required thermal resistance and vapour control layer placement for safe envelope design.
- Energy consumption: the temperature variation dictates the heating and cooling demand. The mild winters in CZ21 suggest a lower heating load compared to CZ66, but the high humidity across all zones means that latent cooling (dehumidification) may be a significant factor in summer energy use.
- Climate zone differentiation: the minor but distinct differences between the zones (e.g., the lower winter temperatures in CZ66) underscore the necessity of performing a zone-specific hygrothermal analysis rather than applying a single design solution across all areas.

## Methods

### Indoor climate calculation under intermittent conditioning

Indoor climate data are fundamental to evaluating hygrothermal and bio-hygrothermal risks in residential buildings under varying conditioning regimes. Although ASHRAE 160, adopted in Australia as AIRAH DA 07, provides widely accepted criteria, it does not address the uncontrolled RH of intermittent conditioning patterns in Australian homes. In practice, the absence of explicit indoor temperature and RH thresholds allows dwellings to operate beyond recommended comfort levels, particularly with uncontrolled RH under intermittent operation. To capture these localised conditions, this study employed the AccuRate simulation tool within NatHERS, applying an intermittent conditioning schedule consistent with the prescribed operating patterns and thermostat settings within the national building regulations (NCC) (see Table 2).

**Table 2.** Intermittent conditioning schedules, NCC 2022, Volume Two, summary of table H6V2a

Hour ending at	Duration (h)	Habitable rooms other than bedrooms	Bedrooms
1:00–6:00	6	**OFF**	ON
7:00–9:00	3	ON	ON
10:00–18:00	9	ON	**OFF**
19:00–23:00	5	ON	ON
0:00	1	**OFF**	ON
Total		On 16 hours, 66.7% of a day	On 14 hours, 58.3% of a day

Bold text highlights those selections included in this study.

AccuRate simulates hourly indoor temperature profiles for each room by incorporating variables such as building layout, wall orientation, room function, construction material and expected occupancy. Interior moisture generation applied the principles of a four-bedroom home, as specified in ASHRAE 160 (Table 3).

**Table 3.** Residential design moisture generation rates from ASHRAE 160, 2016

Number of bedrooms	Number of occupants	Moisture generation rate		
1 bedroom	2	7 L/day	0.8 × 10^−4^ kg/s	0.64 b/h
2 bedrooms	3	9 L/day	1.0 × 10^−4^ kg/s	0.83 b/h
3 bedrooms	4	10 L/day	1.2 × 10^−4^ kg/s	0.92 b/h
**4 bedrooms**	**5**	**11 L/day**	**1.3 × 10^−4^ kg/s**	**1.01 b/h**
Additional bedrooms	+1 per bedroom	+1 L/day	+0.1 × 10^−4^ kg/s	+0.11 b/h

Bold text highlights those selections included in this study.

In the hygrothermal modelling framework, intermittent conditioning was implemented through a time-resolved conditioning boundary condition coupled with an operational schedule. Specifically, the indoor environment was simulated under two alternating states:

- Conditioned periods, during which indoor temperature and humidity were both controlled according to predefined setpoints, representing active operation of heating, cooling and/or dehumidification systems.
- RH not controlled, during which the conditioning system was inactive and indoor RH was not constrained by setpoints. Instead, indoor temperature and RH were computed dynamically based on heat and moisture exchange with the outdoor climate, internal moisture generation and transient hygrothermal processes within the building envelope.

This implementation enables the model to capture transient cyclic behaviours, including moisture accumulation during controlled and uncontrolled RH periods and partial drying during both periods. The resulting time-dependent indoor boundary conditions were directly coupled with the hygrothermal simulation of the building envelope to assess moisture response and mould growth potential under realistic residential operating conditions. This approach also allows the simulation to represent dynamic fluctuations in indoor RH under typical Australian residential conditions, including internal moisture generation associated with occupant activities and the intermittent operation of air-conditioning systems.

The indoor climate profiles calculated from AccuRate software are used as critical input for WUFI ©Pro 6.0 and WUFI©VTT [33,34], transient simulation tools employed for the assessment of heat and moisture transport through building envelopes and the prediction of mould growth risk. An uncontrolled RH intermittent conditioning scenario is modelled based on the profiles derived from AccuRate results, while indoor temperature is maintained within controlled limits (≤21.1 °C) and RH can reach up to 100%.

### Construction requirements of different climate zones

This study selected NatHERS CZ21, CZ64 (+CZ62) and CZ66 representing three distinct climatic zones within south-eastern Australia. The NCC establishes only three well-populated CZs, whereas NatHERS includes nine finer thermal zones within the areas bounded by this study. The construction material choices for the hygrothermal and bio-hygrothermal simulations in this study were modelled to align with the NCC climate classification requirements. This ensures that the results are directly relevant to the regulatory framework for residential building design and construction in Australia. Accordingly, the base case design incorporates construction details such as compliant external pliable membranes, in line with Standards Australia AS 4200.1: pliable building membranes and underlays [43,44], as shown in Table 4. In doing so, the study strengthens both the regulatory applicability of its findings and their practical relevance to building performance assessment under realistic moisture exposure conditions.

**Table 4.** 7-Star regulatory water vapour diffusion requirements of exterior membranes, NCC 2022

NCC climate zone	Required exterior membranes	Vapour permeances		Corresponding water vapour diffusion resistance factor (these values assume the layer thickness is 1 mm)	Corresponding water vapour diffusion resistance factor applied in this study
		Min ≥ (μg/N·s)	Max < (μg/N·s)		
CZ 1–3	No requirements				
**CZ 4–5**	**Min Class 3**	**0.1429**	**1.1403**	**≥175.4078 to <1399.5801**	**1398.0**
**CZ 6–8**	**Min Class 4**	**1.1403**	**No max**	**<175.4078**	**175.4**

Bold text highlights those selections included in this study.

In NCC-CZ4, CZ6 to CZ8, clay masonry veneer external wall systems are required to incorporate a ventilated and drained cavity between the brickwork and the supporting timber or steel frame. A portion of wind-driven rain typically penetrates the clay masonry veneer and enters this cavity. The cavity, also referred to as an air gap or air space, is typically 40 mm wide, allowing both ventilation and drainage to occur. This arrangement enhances durability by minimising moisture transfer to the structural frame [44], as shown in Fig. 4.

**Figure 4:**
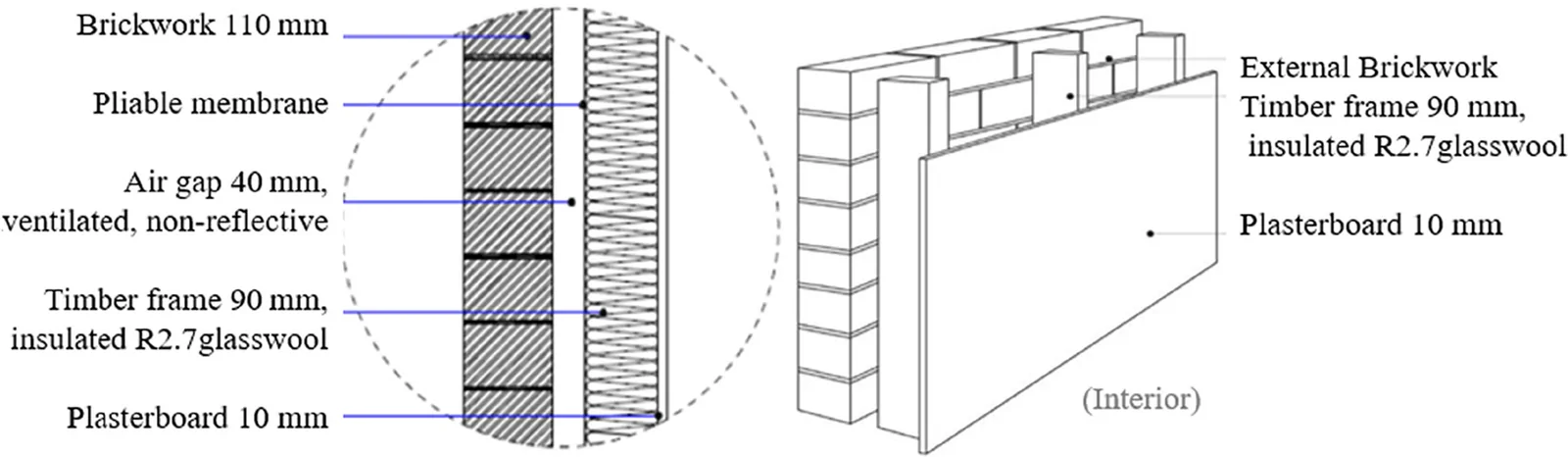
Section and schematic diagram of clay masonry veneer external wall system 2003–2025 (left: wall section, right: corresponding schematic).

### Parameters of hygrothermal simulation

In this study, strategically positioned monitoring points were used to evaluate how both indoor and outdoor conditions influence the thermal and moisture behaviour of the wall assembly under intermittent conditioning. A default air exchange rate (ACH) of 0.2 was applied, representing typical indoor ventilation conditions in recent Australian 7-star energy-efficient dwellings [45]. This value corresponds to room-level air exchange and does not represent infiltration within specific building envelope layers or wall assemblies. Each wall was simulated over a 10-year period to capture thermal performance, moisture content and changes in water content, highlighting potential condensation and mould growth under uncontrolled RH conditions. Material properties for each layer, including vapour permeability, thermal conductivity and moisture storage, were assigned based on manufacturer data and standard material libraries (see Table 5).

**Table 5.** Material properties applied in this study

Material/construction layer	Layer thickness (m)	Bulk density (kg/m^3^)	Porosity (m^3^/m^3^)	Specific heat capacity (J/kg·K)	Thermal conductivity (W/mK)	Water vapour diffusion resistance factor
Fibre cement sheathing board	0.006	1380	0.479	840	0.5	127.7
Extruded clay brick	0.11	1820	0.41	959	0.6	9.5
External membrane	0.001	130	0.001	2300	2.3	175.4
Glass-wool batt insulation	0.09	26.2	0.978	1650	0.036	1.8
Interior vapour control layer	0.001	85	0.086	2500	2.4	34,000
Paper-faced gypsum plaster board	0.010	880	0.65	1050	0.163	6

Key inputs for the hygrothermal simulations include climatic data, material properties, ventilation rates and strategically positioned monitoring points within the wall assemblies. To evaluate mitigation strategies, the study examines the use of ventilated cavities and an interior vapour control layer, both individually and in combination, under uncontrolled RH intermittent conditioning patterns. Figure 5 illustrates the comprehensive layering applied to each wall system and the monitoring positions in the WUFI Pro software utilised in the research.

**Figure 5:**
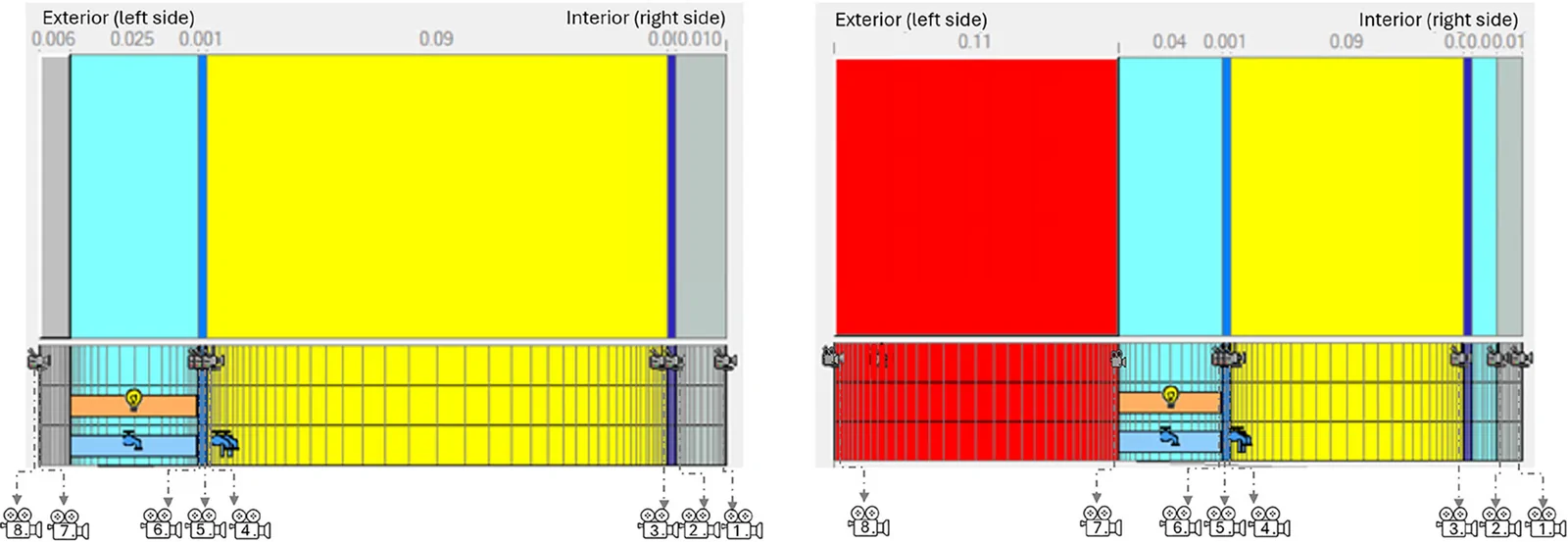
WUFI monitoring-point settings applied to all layers, CFCS (left) and clay masonry veneer (right) clad external wall systems.

In the WUFI simulation, the monitoring point was placed at a selected layer/interface within the wall assembly to capture hygrothermal conditions at a critical location of interest. These positions are used to evaluate transient temperature and RH development within the assembly, which is essential for assessing moisture accumulation and mould growth risk. Eight monitoring points were systematically placed across four critical layers in each wall assembly to capture bio-hygrothermal performance and moisture dynamics at key material interfaces:

- interior and exterior surfaces of the plasterboard (camera 1 and camera 2)
- interior and exterior surfaces of the glass-wool batt insulation (camera 3 and camera 4)
- interior and exterior surfaces of the pliable membranes (camera 5 and camera 6)
- interior and exterior surfaces of the cladding (camera 7 and camera 8)

The effect of these strategies on internal moisture buffering and temperature moderation was assessed to determine their suitability for high-performance building envelopes subjected to intermittent, uncontrolled temperature and RH conditions.

Given the significance of moisture-related challenges in timber-framed construction, this study focuses on the point immediately adjacent to the interior surface of the exterior water vapour control layer next to the timber frame and insulation, which has consistently exhibited the greatest risk in previous research [36,37,46].

### Bio-hygrothermal mould growth prediction

This study applies bio-hygrothermal simulation to assess the risk of interstitial mould growth within building envelopes, using the Mould Growth Index (MI) as a quantitative metric to predict mould proliferation based on environmental and material characteristics. The WUFI VTT add-on software was used to post-process the hygrothermal simulation results to predict mould growth risk [47–49]. The enhancement strategies of lower water vapour diffusion resistivity of the exterior weather resistive layer, the addition of a ventilated cavity and the addition of an interior vapour control layer were assessed within the context of the calculated MI. According to NCC 2022 Volume Two [44], the condensation verification approach requires that MI values do not exceed 3.0 at either:

- the interior surface of the water control layer, or
- any building fabric surface located interior to the water control layer.

The analysis employs the VTT MI model to predict mould formation risk over time on a scale from 0 (no growth) to 3 (visible surface mould covering less than 10% or microscopic coverage less than 50%) and 6 (extensive surface colonisation, up to 100% coverage visually). This empirical model incorporates variations in substrate material, surface temperature and RH to estimate both the accumulation and decline of mould on an hourly basis. The study evaluates mould risk over short-term (seasonal) and long-term (annual) periods, providing a comprehensive assessment of cyclic and sustained hygrothermal exposure on studied wall systems. As this study was focused on the predominance of plantation softwood-based timber framing, the mould sensitivity class of very sensitive was selected.

## Results

This section presents results under the intermittently controlled RH conditioning. During operational periods, the indoor temperature was maintained below 21.1 °C, with the RH regulated to a maximum of 70% in accordance with ASHRAE 160 guidelines. Conversely, during unconditioned periods, the temperature control remained capped at 21.1 °C, while the indoor RH floated dynamically, governed by ambient outdoor climate variations and occupant density.

### Indoor climate profiles

The indoor climate is a critical boundary condition for hygrothermal simulation of external wall systems, serving as one of the primary drivers of moisture transport and heat transfer through the building envelope. It also reflects the indoor conditioning pattern. During the heating season, the indoor climate determines the ‘warm side’ moisture load, driving vapour into walls, influencing condensation where dew points occur and affecting mould growth risk when surface or interface humidity exceeds critical thresholds. Hygrothermal simulations (such as WUFI) model the coupled flow of heat and moisture and therefore require both outdoor and indoor climate data to accurately assess potential risks, including condensation and mould growth.

#### The RH results of CZ21

One of the key objectives of this research is to identify the rooms most susceptible to moisture accumulation and mould growth under an intermittent RH conditioning regime. Furthermore, it examines how variations in indoor RH arise from the combined effects of floor plan layout, building orientation and the primary activities conducted within each space. Note that shower moisture and latent heat loads are currently excluded from the AccuRate simulation.

Figure 6 compares the indoor RH profiles between intermittent RH conditioning and continuous conditioning for NatHERS CZ21 as an example. The horizontal axis represents hours; the vertical axis represents RH. The blue drop indicates the 70% threshold, which is the prescribed limit under ASHRAE 160. Compared across the three rooms, the bedroom shows the highest RH.

**Figure 6:**
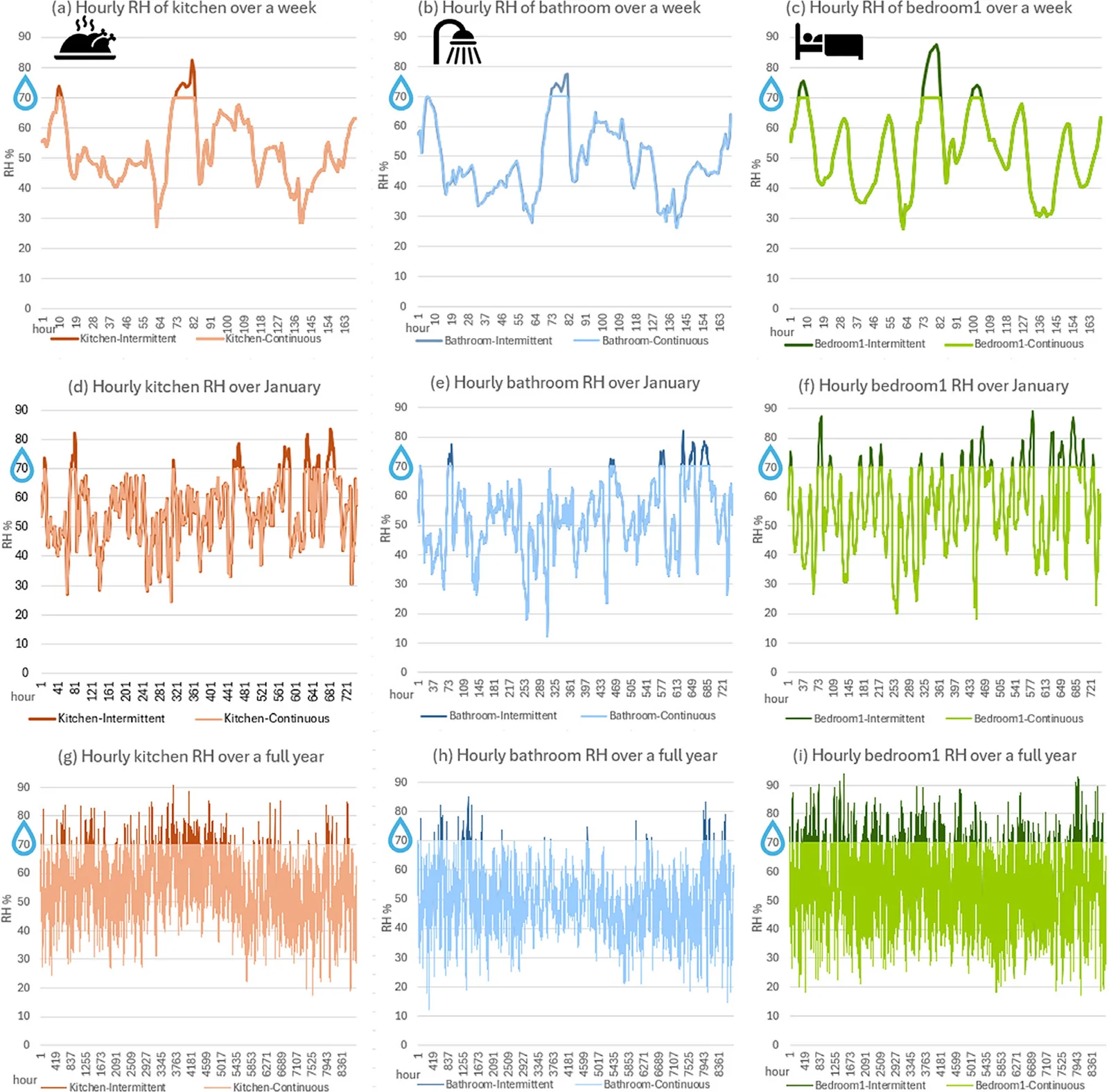
RH comparison between two conditioning scenarios for the north-oriented house in CZ21.

To gain an overall understanding of the RH behaviour, the key measurements are presented. Table 6 presents the key statistical measures of the annual simulated indoor temperature profiles for different rooms in two plan orientations located within NatHERS CZ21. For each room, maximum, minimum and average temperatures are presented alongside the percentage of times when the RH was above 70%, the threshold typically associated with thermal comfort and mould growth risk limits prescribed within ASHRAE 160 and AIRAH DA07.

**Table 6.** Key statistical measures of RH intermittent conditioning in CZ21 (%)

Climate	Plan orientation	Zone/room	Wall orientation	Max. RH	Min. RH	Avg. RH	RH > 70%
CZ64 (NCC-CZ6)	North 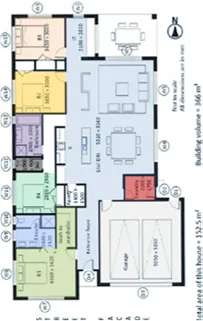	Outdoor		100	7	64.6	+39.5%
		Kitchen, living	N, S, E	95.5	20.7	53.6	+8.7%
		Bathroom	W	85.0	10.5	49.1	+3.4%
		Bed 1	W, S	93.4	13.4	54.9	+14.7%
		Bed 2	W	92.5	14.3	53.5	13.1%
		Bed 3	N, W	93.0	13.6	53.1	+14.0%
		Bed 4	W	92.8	14.2	53.7	+13.2%
		Laundry	E	86.5	10.4	50.3	+4.2%
		En-suite	W	85.4	12.8	48.7	+3.2%
		Toilet	W	84.0	7.3	48.3	+2.9%
	East 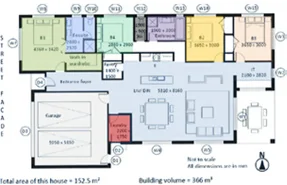	Kitchen, living	S, E, W	90.6	19.3	54.2	+8.6%
		Bathroom	N	92.6	16.3	52.7	+12.7%
		Bed 1	W, N	93.4	1.9	54.0	+14.2%
		Bed 2	N	92.6	16.3	52.7	+12.7%
		Bed 3	N, E	93.1	14.7	52.4	+12.8%
		Bed 4	N	92.9	16.0	52.8	+12.8%
		Laundry	S	86.7	10.4	51.1	+4.6%
		En-suite	N	85.3	14.4	48.2	+3.2%
		Toilet	N	84.5	15.4	47.3	+2.9%

Shaded cells highlight outdoor zones.

Of all rooms and orientations of CZ21 under intermittently controlled RH conditioning, indoor RH varied substantially across different functional zones and orientations. In the north-facing plan, the kitchen and living area reached a maximum RH of 95.5%, with the RH exceeding 70% for approximately 8.7% of a full year period, while bedrooms showed RH values above 70% for 13%–15% of the time, reflecting higher moisture retention in these zones. Wet areas such as the bathroom and laundry experienced high RH peaks (85.0%–86.5%) but shorter periods above 70% (3%–4%), indicating lower total moisture loads.

In the east-facing plan, a similar trend was observed, with bedrooms exhibiting sustained periods of RH above 70% (12.7%–14.2%), highlighting their susceptibility to elevated moisture loads under intermittent conditioning. Wet zones again showed high peak RH but limited duration above critical thresholds. Notably, the en-suite and toilet zones maintained comparatively lower average RH (47%–48%) and short durations above 70%, consistent with lower moisture generation or more effective ventilation. These results indicate that intermittent indoor conditioning produces significant variability in RH across building zones, with bedrooms consistently experiencing the highest sustained humidity levels. This reflects findings from residential building surveys where the bedrooms often show the greatest presence of interior surface mould [46].

#### The RH results of CZ64 (+CZ62)

The results presented in Table 7 summarise key statistical measures of indoor RH under uncontrolled RH intermittent conditioning for a typical CZ64 dwelling located in NCC-CZ6. The data are disaggregated by plan orientation (north and east), individual rooms or zones and wall orientations, providing maximum, minimum and average RH values, as well as the percentage of time the RH exceeds 70%.

**Table 7.** Key statistical measures of RH intermittent conditioning in CZ64 (%)

Climate	Plan orientation	Zone/room	Wall orientation	Max. RH	Min. RH	Avg. RH	RH > 70%
CZ64 (NCC-CZ6)	North 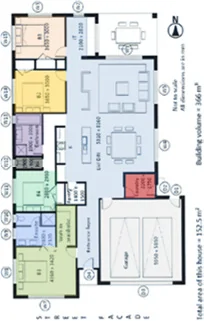	Outdoor		100	10	74.1	+64.1%
		Kitchen, living	N, S, E	99.7	18.6	58.7	+17.3%
		Bathroom	W	92.9	15.2	55.9	+10.3%
		Bed 1	W, S	99.6	19.9	59.3	+21.4%
		Bed 2	W	99.0	15.2	58.0	+19.6%
		Bed 3	N, W	99.5	17.9	57.8	+20.3%
		Bed 4	W	99.1	14.9	58.2	+19.8%
		Laundry	E	94.6	17.0	57.5	+13.0%
		En-suite	W	93.6	18.1	54.8	+9.5%
		Toilet	W	90.5	14.3	54.6	+7.9%
	East 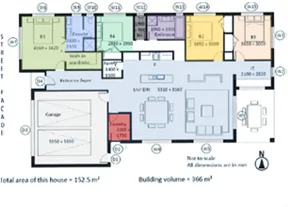	Kitchen, living	S, E, W	99.6	18.6	59.4	+17.5%
		Bathroom	N	93.1	18.4	53.9	+7.9%
		Bed 1	W, N	99.6	14.7	58.6	+21.1%
		Bed 2	N	98.9	19.1	57.6	+19.2%
		Bed 3	N, E	99.2	17.0	57.5	+19.5%
		Bed 4	N	99.4	19.6	57.7	+19.6%
		Laundry	S	95.3	18.1	58.2	+14.1%
		En-suite	N	93.7	17.9	54.4	+9.6%
		Toilet	N	91.2	18.4	53.9	+7.9%

Shaded cells highlight outdoor zones.

For the north-oriented plan, outdoor RH ranges from 10% to 100%, with an average of 74.1% and 64.1% of the time exceeding 70%. Indoor RH levels are generally lower, with the highest average RH observed in Bed 1 (59.3%) and the lowest in the en-suite (54.8%). Maximum RH values approach saturation in all rooms, particularly in the bedrooms and kitchen/living (KL) areas, which reach up to 99.7% RH. The proportion of time exceeding 70% RH varies substantially across spaces, with bedrooms consistently showing prolonged exposure above this threshold (Bed 1: 21.4%, Bed 2: 19.6%, Bed 3: 20.3%, Bed 4: 19.8%), while service spaces such as the bathroom and laundry show lower percentages (bathroom: 10.3%, laundry: 13.0%).

In the east-oriented plan, a similar trend is observed. Maximum RH values remain close to 100% for most across the majority of rooms, with the highest values recorded in Bed 1 (99.6%) and slightly lower values in the bathroom (93.1%). Average RH levels range from 53.9% in the toilet to 59.4% in the KL area. RH exceedance above 70% follows a comparable pattern to the north orientation, with bedrooms again showing the highest values (Bed 1: 21.1%, Bed 2: 19.2%, Bed 3: 19.5%, Bed 4: 19.6%) and service spaces remaining lower (bathroom: 7.9%, laundry: 14.1%).

Overall, the data indicate that under uncontrolled RH intermittent conditioning, all indoor zones experience periods of high RH, with bedrooms consistently showing both high peak RH and extended rates above 70%. This suggests that these spaces may be at greater risk of moisture accumulation and potential mould growth. The kitchen and living areas also exhibit prolonged high RH, whereas bathrooms, laundries and toilets generally experience lower average RH and reduced exposure time above 70%. These results highlight the influence of room function and wall orientation on indoor hygrothermal conditions.

#### The RH results of CZ66

The data in Table 8 present key statistical measures of indoor RH in CZ66, NCC-CZ4 and CZ6. The data are organised in the same way as previous tables: the maximum, minimum and average RH, as well as the percentage of time the RH is >70%.

**Table 8.** Key statistical measures of RH intermittent conditioning in CZ66 (%)

Climate	Plan orientation	Zone/room	Wall orientation	Max. RH	Min. RH	Avg. RH	RH > 70%
CZ66 NCC-CZ4 & CZ6	North 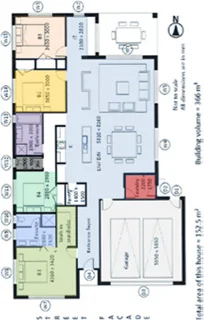	Outdoor		100	7	75.5	+64.1%
		Kitchen, living	N, S, E	100	18.6	55.6	+23.3%
		Bathroom	W	100	13	55.4	+13.4%
		Bed 1	W, S	100	14.8	57.0	+14.4%
		Bed 2	W	100	13	55.4	+18.1%
		Bed 3	N, W	100	14.2	54.9	+18.8%
		Bed 4	W	100	10.5	55.6	+17.9%
		Laundry	E	100	12.1	56.0	+12.4%
		En-suite	W	100	13.31	51.4	+5.7%
		Toilet	W	98.1	8.7	53.0	+6.1%
	East 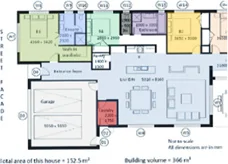	Kitchen, living	S, E, W	100	15.8	56.3	+10.6%
		Bathroom	N	100	12.7	53.3	+7.6%
		Bed 1	W, N	100	13.0	56.0	+19.4%
		Bed 2	N	100	15.4	54.7	+17.5%
		Bed 3	N, E	100	14.3	54.5	+17.4%
		Bed 4	N	100	15.6	54.7	+17.7%
		Laundry	S	100	12.1	57.1	+14.2%
		En-suite	N	100	14.3	50.8	+5.5%
		Toilet	N	95.7	14.7	51.6	+5.5%

Shaded cells highlight outdoor zones.

For the house with a north-facing orientation, the maximum RH was consistently 100% across most indoor spaces. The minimum RH values for indoor rooms ranged from 10.5% (Bed 4) to 18.6% (kitchen and living area). The kitchen and living area registered the highest average RH at 55.6% and the longest duration above 70% at +23.3%. Bed 2 and Bed 3 followed closely, with average RH values of 55.4% and 54.9%, and corresponding exceedance percentages of +18.1% and +18.8%. Rooms such as en-suite and toilet demonstrated the lowest average humidity, with the en-suite recording the absolute lowest average RH at 51.4% and the shortest duration above 70% at +5.7%.

The east-facing house demonstrated similar overall trends for RH, with maximum RH values also predominantly 100% across all rooms. The minimum RH values were slightly less dispersed than in the north, ranging from 12.1% (laundry) to 15.8% (kitchen and living area). The highest average RH under plan east was observed in the laundry at 57.1%, which also exhibited the longest duration above 70% at +14.2%. The kitchen and living area had the second-highest average RH at 56.3% and an exceedance of +10.6%. Among the bedrooms, Bed 1 and Bed 4 recorded the highest average RH at 56.0% and 54.7%, with exceedance percentages of +19.4% and +17.7%, respectively, suggesting high-humidity loads in these spaces. Conversely, the en-suite and toilet again showed the lowest average humidity values, with the en-suite registering the minimum average RH at 50.8% and the toilet exhibiting the lowest exceedance percentage at +5.5%.

### The hygrothermal results

The impact of RH variations caused by different orientations and conditioning regimes on moisture accumulation within the different external wall system components was then analysed. Given that each room type serves a different function and is influenced by the orientation of adjacent external walls, the indoor climate of each room was individually simulated. The overall results indicate that the monitoring point located on the interior side of the exterior membrane shows the highest risk of occurrence; therefore, this paper focuses on that point for further analysis.

Table 9 presents the simulated final water accumulation results (in kg/m^2^) of the clay masonry veneer external wall system within assessed CZs. The analysis commenced with a uniform initial water content of 1.38 kg/m^2^ in various assemblies. The first three columns on the left are plan orientation, wall orientation and room names. The primary variables investigated include four distinct climates (CZ21, CZ64, CZ66 of NCC-CZ6 and CZ66 of NCC4) and the presence or absence of an interior vapour control layer, as listed in each column in purple.

**Table 9.** Hygrothermal performance of clay masonry veneer wall system

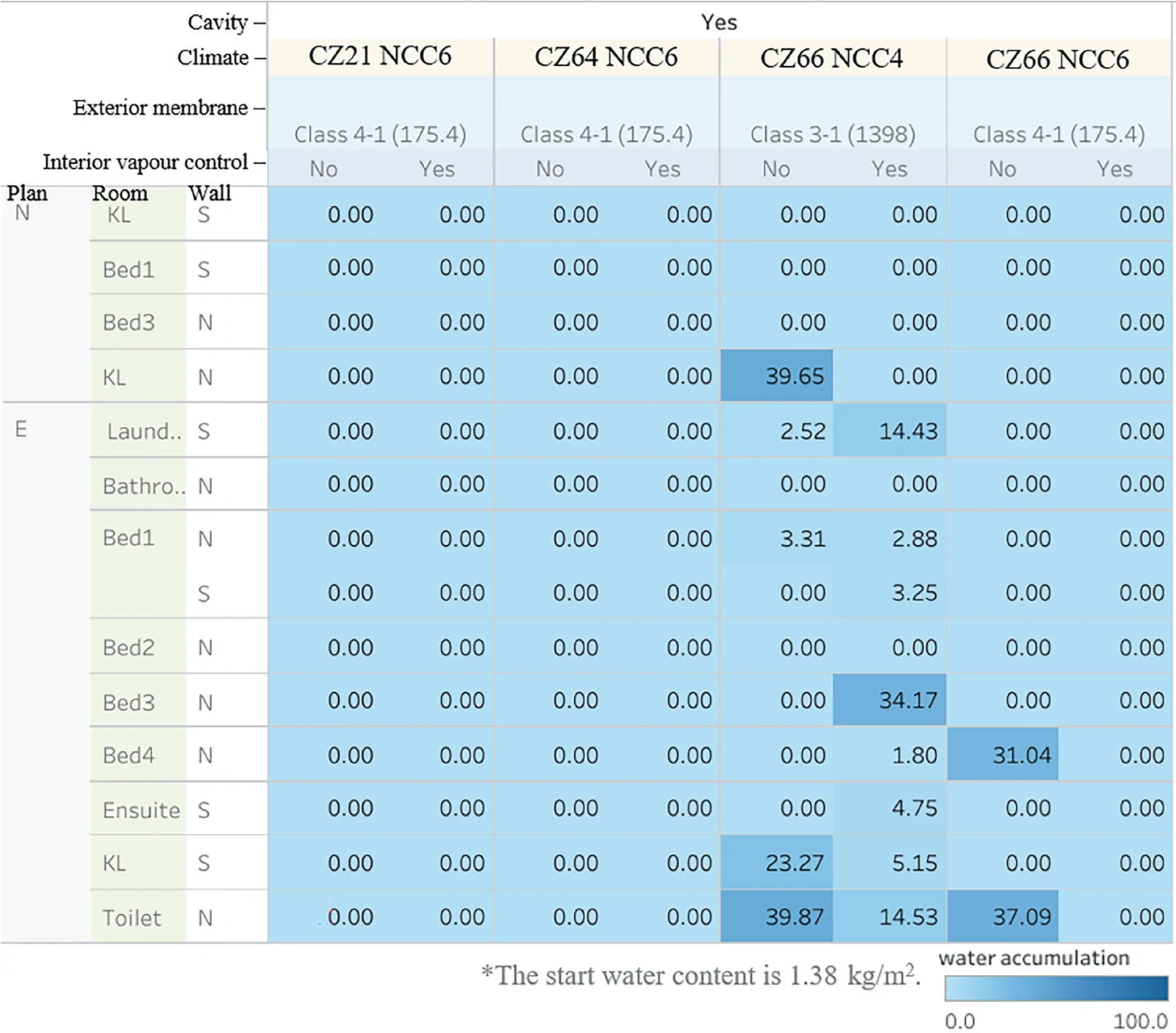

The results indicate that for scenarios in CZ21 and CZ64, no moisture accumulation occurred in any of the tested wall configurations, with cavity applied to all, regardless of the presence of an interior membrane. The final water content in these cases remained at or below the initial value. The legend on the top left indicates the level of moisture accumulation, and the darker colour means higher water content.

In contrast, water accumulation was observed under CZ66 NCC-CZ4 and CZ66 NCC-CZ6, with this behaviour being predominantly associated with the presence of an interior vapour control layer. When an interior membrane was included (the ‘Yes’ columns), water accumulation was eliminated in NCC-CZ6 across all rooms and orientations. However, the influence of the interior vapour control layer was not uniform across CZs. In NCC-CZ4, a few cases exhibited moisture accumulation when an interior vapour control layer was applied compared with cases without one. For instance, in the south-facing laundry wall under CZ66 NCC-CZ4, water accumulation increased from no accumulation without an interior vapour control layer to 14.43 kg/m^2^ with its inclusion. Similarly, in the north-facing Bed 3 wall of the east-oriented plan, accumulation increased from 0.00 kg/m^2^ to 34.17 kg/m^2^ following the addition of the interior vapour control layer.

The highest levels of water accumulation were recorded in both house plans north-facing and east-facing, without an interior membrane. Under the CZ66 NCC-CZ4 scenario, the north-facing walls of the KL and toilet rooms exhibited the most substantial moisture content, reaching 39.65 kg/m^2^ and 39.87 kg/m^2^, respectively. Under the CZ66 NCC 6 scenarios, for the east-facing plan, the north-facing walls of the toilet and Bed 4 demonstrated the highest accumulation, with values of 37.09 kg/m^2^ and 31.04 kg/m^2^, respectively. These results indicate a pronounced and consistent influence of specific climatic exposures coupled with interior and exterior vapour control membranes on interstitial moisture accumulation. Here, further studies would be required to understand the inconsistent performance of the applied interior vapour control layer and to investigate alternatives, such as vapour control layers with varying or humidity-controlled permeances.

Table 10 presents the hygrothermal analysis to assess the performance of CFCS wall assemblies, which commenced with an initial water content of 1.48 kg/m^2^. The structure of the table is similar to the previous one but includes the additional aspect of a ventilated cavity, which is currently not required within the Australian building regulations. The results are presented in two primary configurations, distinguished by the presence or absence of cavity and interior vapour control layer. The dotted line in the middle separates the panel with no cavity on the left from the panel with cavity on the right.

**Table 10.** Hygrothermal performance of CFCS wall systems

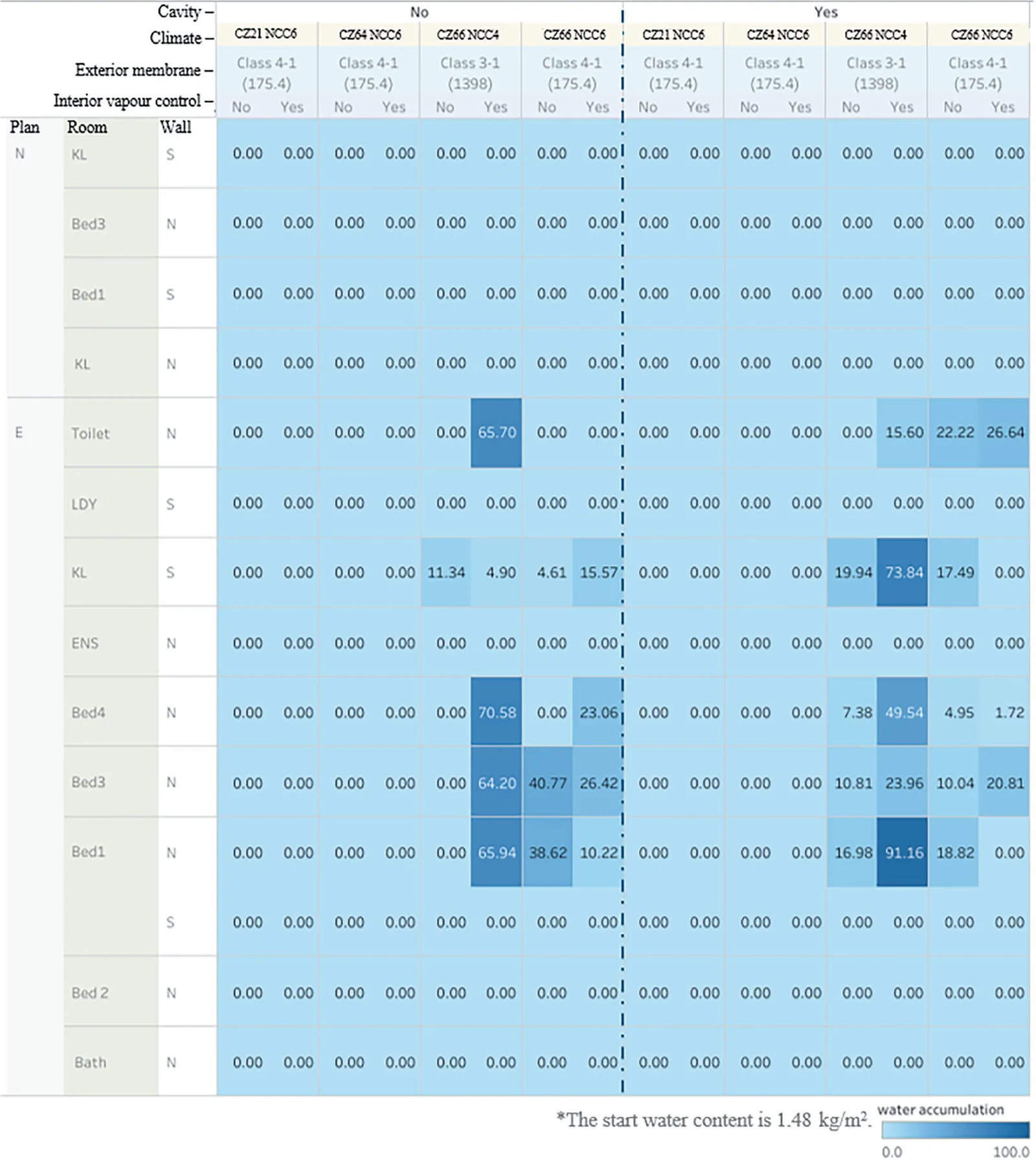

Consistent with the previous findings, the CFCS wall assemblies demonstrated no accumulation of water in CZ21 and CZ64 inside the wall. However, in the more demanding CZ66 NCC-CZ4 and CZ66 NCC-CZ6 climates, the CFCS walls exhibited a pronounced vulnerability to interstitial moisture accumulation, which is greater than in the previously analysed clay masonry veneer system.

In the first primary configuration (‘No cavity’), substantial water accumulation was recorded when the interior membrane was absent. The highest values were observed in the north-facing walls of the east-oriented house under the CZ66 NCC-CZ4 scenario, reaching 70.58 kg/m^2^ in Bed 4 and 65.94 kg/m^2^ in Bed 1. To contextualise this magnitude, such values reflect severe liquid-phase condensation rather than standard vapour sorption, approaching near-saturation within the hygroscopic layers at the external sheathing interface. In typical dry lightweight timber-framed walls, baseline hygroscopic equilibrium rarely exceeds 15–20 kg/m² [50]. Furthermore, the inclusion of an interior control layer with a Class 4 exterior membrane mitigated this effect but increased in some cases if the Class 4 membrane was replaced by a less permeable Class 3 exterior membrane. However, there are also exceptions; for example, when the house is oriented east, the moisture content in the walls of the toilet, Bed 4, Bed 3 and Bed 2 increased from an initial amount of 1.48 kg/m^2^ to 65.70, 70.58, 64.20 and 65.94 kg/m^2^. European and international hygrothermal criteria restrict cumulative winter interstitial condensation to a maximum permissible limit of 1.0 kg/m² for general interfaces (and 0.5 kg/m² for non-absorbent interfaces). Values on the order of 70–91 kg/m² exceed this limit by orders of magnitude, indicating severe systemic water logging [51].

The second primary configuration (‘Yes’ – with cavity) exhibited even higher levels of moisture accumulation in all cases. The peak moisture content of the entire study occurred under this configuration: a north-facing wall in Bed 1 under CZ66 NCC-CZ4 reached 91.16 kg/m^2^ with an interior membrane. Similar to the no-cavity case, a Class 3 exterior membrane combined with an interior control layer tended to increase moisture content. When both the primary component and the interior membrane were present, the use of a Class 4 exterior membrane showed improvements in some cases. For example, under CZ66 NCC-CZ6, the east-facing KL wall (17.49 kg/m^2^), the south-facing KL wall and the north-facing toilet wall (26.64 kg/m^2^) all demonstrated reduced water accumulation. However, a few exceptions were identified again: specifically, the east-facing toilet wall and Bed 3 wall showed increased moisture when both a Class 4 exterior membrane and an interior membrane were applied. Overall, the performance of the interior membrane remains complex and inconsistent. These findings indicate that, for CFCS assemblies in these climates, the risk of interstitial condensation remains high, particularly when multiple or unsuitable control layers are included, with the interior vapour control layer contributing to variable outcomes.

### The bio-hygrothermal results – mould growth

To ensure consistency with previously validated indoor mould risk simulation studies, the MI system was adopted as the reference framework for assessing mould growth potential. In accordance with NCC 2022, an MI value greater than 3.0 is deemed unacceptable on the interior surface of the water control layer or on any building components located inward of this layer within roofs or external walls. This threshold, also defined by AIRAH DA07, is essential for compliance with Performance Requirements F8P1 (Volume One) and H4P7 (Volume Two) and is critical for minimising the adverse health impacts of water vapour and condensation.

The MI scale from the VTT model [50], used for empirical validation, is defined as:

- 0 < MI ≤ 1: small amounts of microscopic mould growth detectable on surfaces.
- 1 < MI < 3: several localised colonies of microscopic mould growth present on surfaces, potentially visible as mould covering less than 10% of the area, or up to 50% coverage at the microscopic level.
- 3 ≤ MI < 6: visible mould growth with high colonisation, covering more than 50% of the surface area.
- MI = 6: very high and dense colonisation, with approximately 100% surface coverage (maximum rating in WUFI VTT).

Tables 11 and 12 present the results for the MI. The legend in the top-left corner displays the MI values using three distinct colour scales: green indicates an MI <1.0, grey indicates an MI of 1.0 to <3.0 and red indicates an MI ≥3.0. To be clear, green and grey are both acceptable within NCC 2022 whereas red indicates a non-compliant value; further exploration is required. However, international research is increasingly recommending a simulated MI of <1.0, providing the reasoning for the three classifications shown in the table.

**Table 11.** Bio-hygrothermal performance of clay masonry veneer wall system

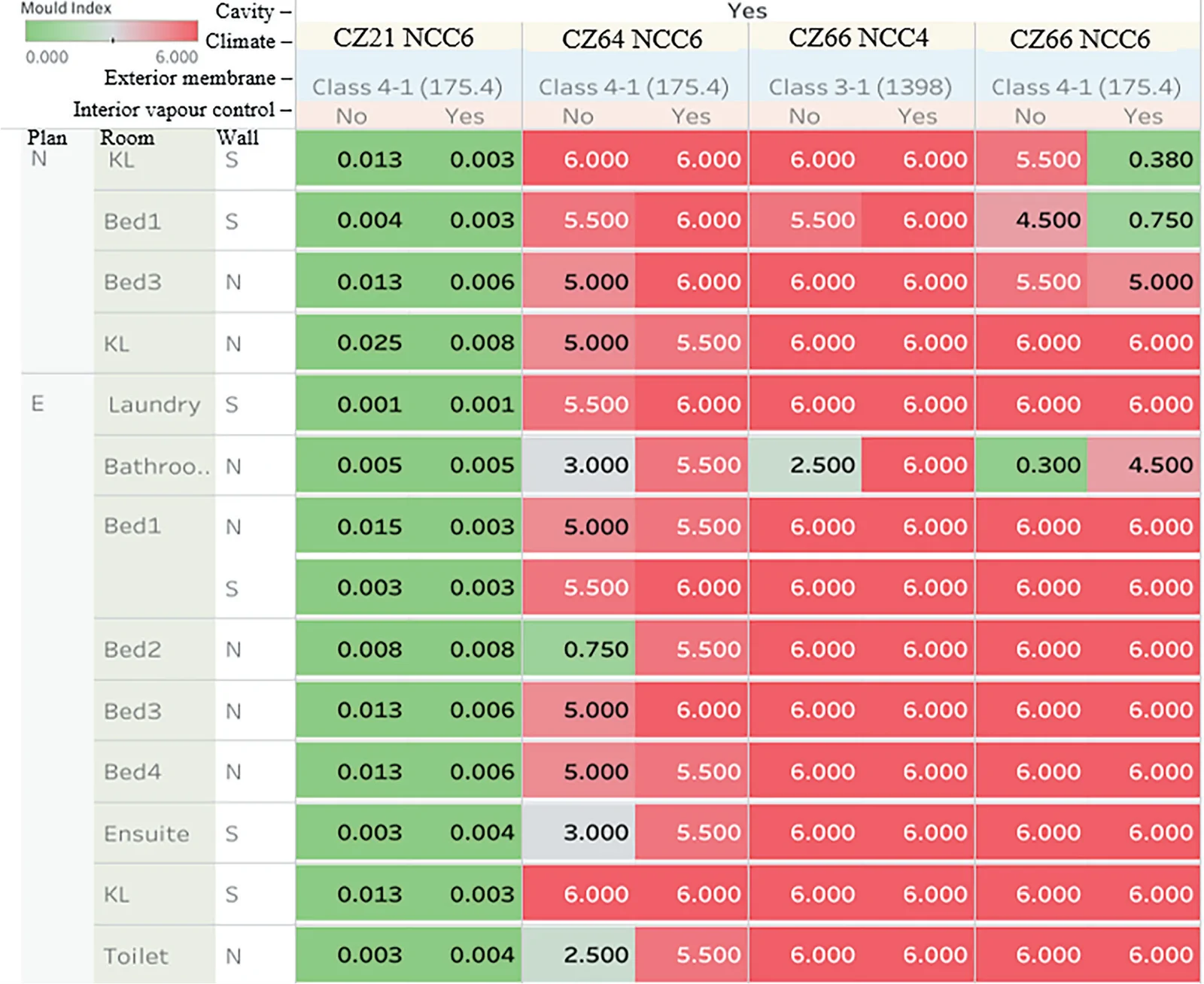

**Table 12.** Bio-hygrothermal performance of CFCS wall system

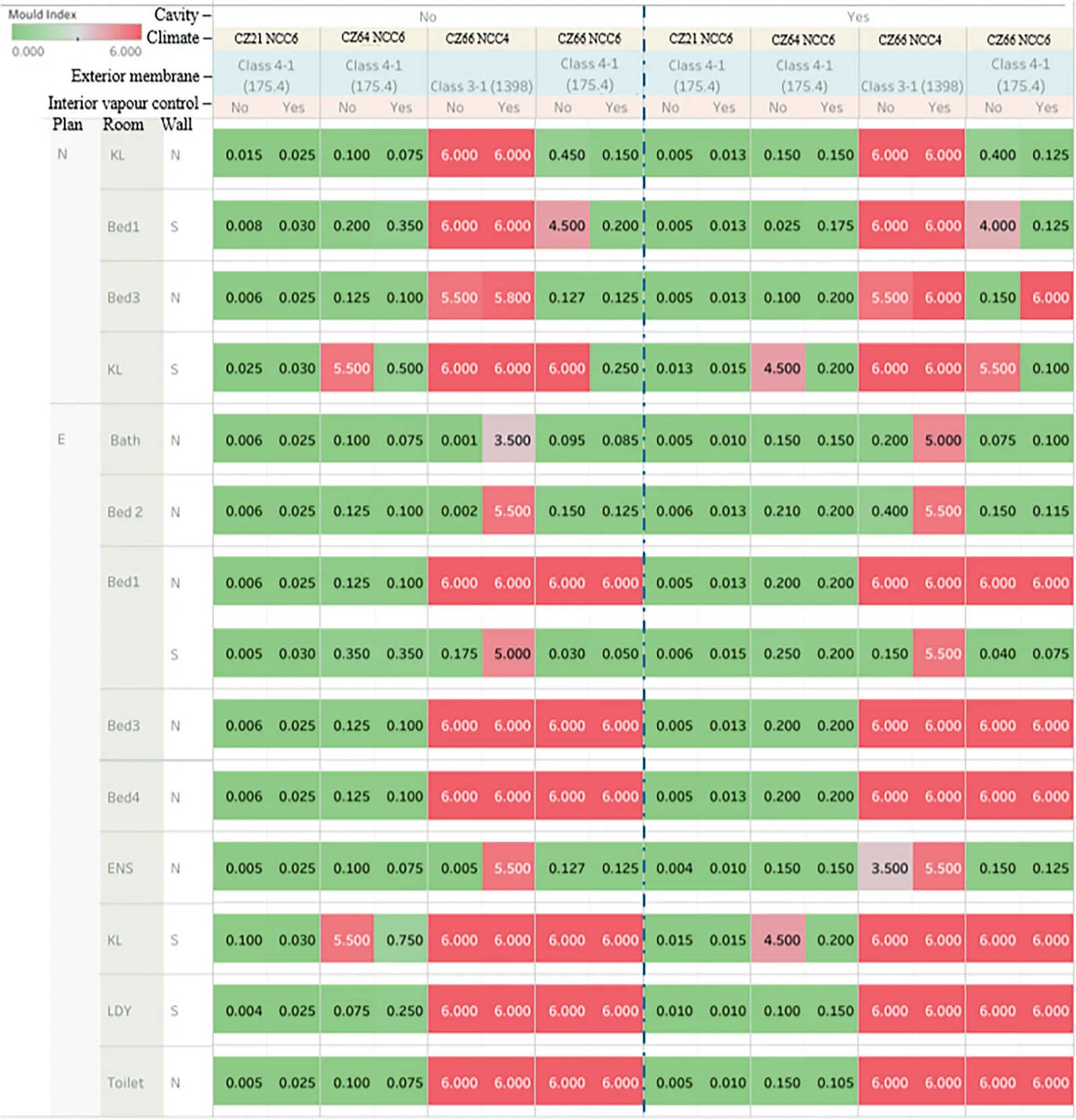

For the clay masonry veneer wall system in Table 11, the results for CZ21 show that the MI remains below 1.0. In CZ64, several results yielded an MI of 0.75–6.0; 4 of the 28 cases (14 failed to meet the NCC requirement of 3.0). A key finding is that the application of the interior membrane resulted in a frequently higher MI, as all results fell within the red scale (MI ≥3.0).

In CZ66-NCC 4, an MI of 6 was frequently observed, indicating a consistent failure to comply with the maximum permissible threshold of 3.0. Among the 28 evaluated cases without an interior membrane, only one case achieved an MI of 2.5, while all remaining cases exceeded acceptable MI values. In CZ66-NCC 6 (the last two columns), three out of 28 cases yielded MI values at or below 3.0, whereas the majority ranged between 5.5 and 6.0.

When the interior membrane was introduced, performance inconsistencies were again evident. For the north-oriented house, KL and Bed 1 showed that MI values reduced from 4.5 or 5.5 to within 1.0. In contrast, for the east-oriented house, bathroom assemblies demonstrated worsened outcomes, with MI values ranging from 0.30 to 4.5.

These results indicate that additional design or control interventions are required to enhance system performance within these CZs. Furthermore, as the maximum reportable MI in the WUFI VTT framework is capped at 6, differentiation among cases that may have exceeded this threshold is not possible, thereby limiting the resolution of the analysis in the most severe scenarios.

Table 12 presents the bio-hygrothermal results for the CFCS external wall system, showing varying performance across different CZs. The structure and legend of the table are the same as those of the previous CFCS table. The dotted line in the middle separates the panel with no cavity on the left from the panel with cavity on the right.

In CZ21, all cases were consistently performed within the required parameters, with the MI remaining at or below 3.0 on the green scale. In CZ64, the system largely met the performance requirements, with only four out of 26 cases exceeding an MI of 3.0. Notably, the application of the interior membrane in this zone ensured these cases remained within the green scale, indicating full compliance with the performance requirement. In contrast, the system consistently failed to meet the performance requirements in CZ66, where the majority of cases recorded an MI of 6.0. Neither the introduction of a cavity nor the application of an interior membrane consistently served as effective mitigation strategies. Reductions in MI, and therefore consistent improvements in performance, were not observed uniformly in this CZ.

## Comparison of continuous and intermittent RH conditioning

To better understand these findings, this section compares them with the continuous conditioning scenarios detailed below. Under these benchmark conditions, prescribed by ASHRAE Standard 160 and DA07, indoor temperatures are maintained between 21.1 °C and 23.9 °C, and RH is regulated to ≤70%.

Figure 7 illustrates the transient hygrothermal results for CFCS timber-framed external wall assemblies across multiple south-eastern Australian CZs, room types and two conditioning regimes. The left grey panel presents continuous conditioning, and the right colourful panel displays intermittent RH conditioning performance. Results are shown for baseline NCC-compliant construction and for enhanced assemblies incorporating interior vapour control (Interior M.), a ventilated cavity (C.) and the combined application of both measures (In. M. + C.). MI values range from 0 to 6; no values below 0.001 are reported. Higher MI values indicate greater interstitial mould growth risks. Each cell displays the predicted MI, with colour shading denoting the room type and the labelled number indicating the MI value. Rows represent successive construction enhancements, progressing from baseline NCC-compliant assemblies for CFCS that include interior vapour control, ventilated cavities and combined mitigation strategies for studied climates, respectively. This structure enables direct comparison of conditioning regime impacts as well as the relative effectiveness of individual and combined moisture-control measures.

**Figure 7:**
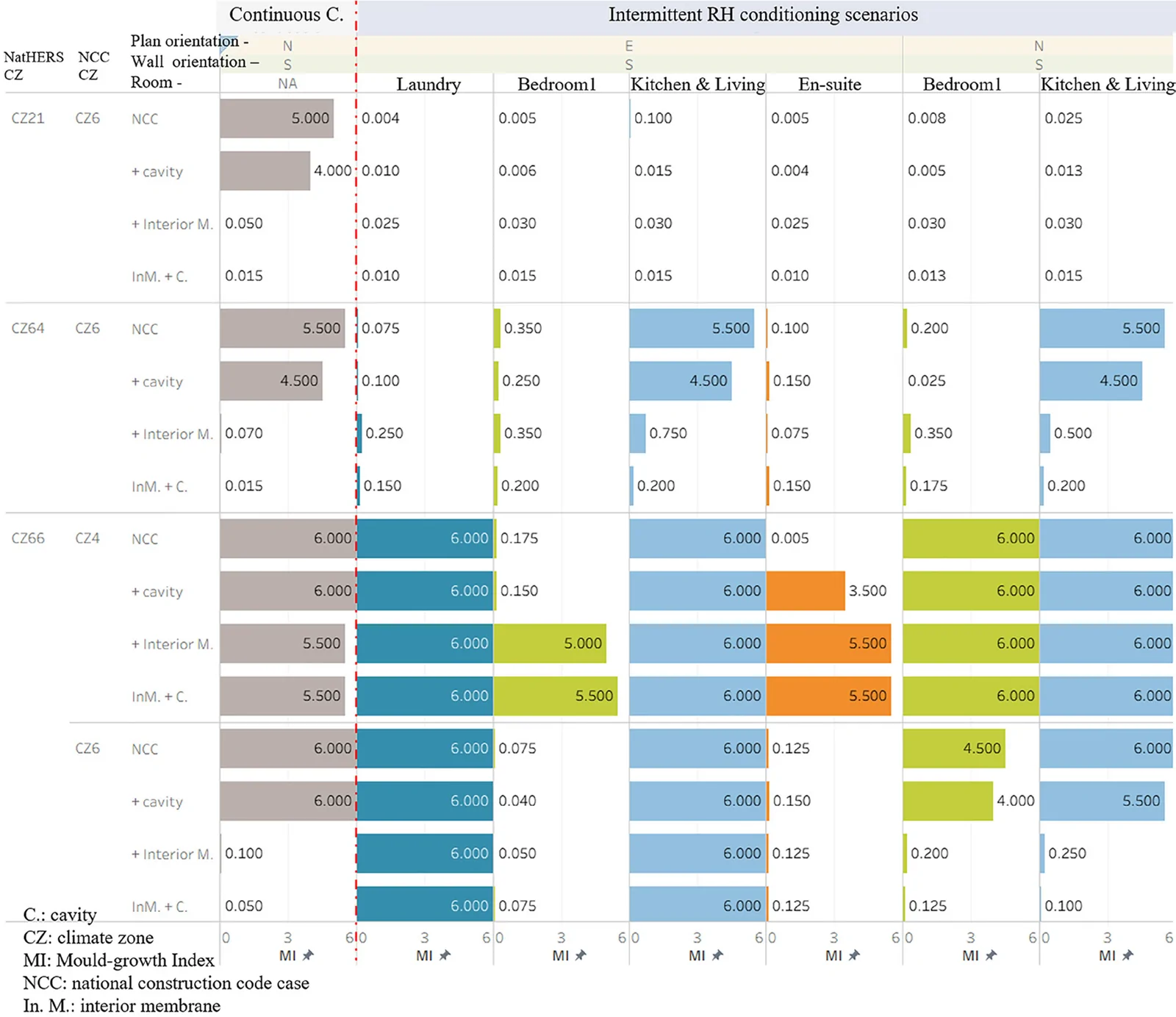
Comparison of bio-hygrothermal results for the CFCS-clad wall under the two studied conditioning regimes.

The intermittent RH conditioning regime generally exhibits lower performance than continuous conditioning but a mixed performance when compared to fully intermittent conditioning scenarios. MI values are typically lower than those observed under fully intermittent conditioning but remain comparable to continuously conditioned cases. Under this regime, construction enhancements result in a clearer stratification of performance, with baseline NCC-compliant assemblies generally achieving MI values below 3.0. However, progressively higher MI values are observed for configurations incorporating interior vapour control (Interior M.), ventilated cavities (C.) and their combined application (In. M. + C.). For CZ66 under NCC-CZ4 conditions, bedrooms and en-suite spaces demonstrate a pronounced increase in MI values, rising from <1.0 for baseline assemblies to approximately 5.5 when all mitigation measures are applied, exceeding the acceptable performance threshold of 3.0 for MI.

As illustrated in Fig. 8, the clay masonry veneer or brick veneer wall system’s performance also varied across CZs. The figure has the same structure as Fig. 7, but fewer variables as the NCC mandates ventilated and drained cavities for clay masonry veneer walls, and the interior vapour control layer is the only intervention applied. In CZ21, the system consistently achieved an MI below the 3.0 threshold. These results were comparable to the ASHRAE-prescribed MI values, indicating no numerically significant difference between the two conditioning patterns.

**Figure 8:**
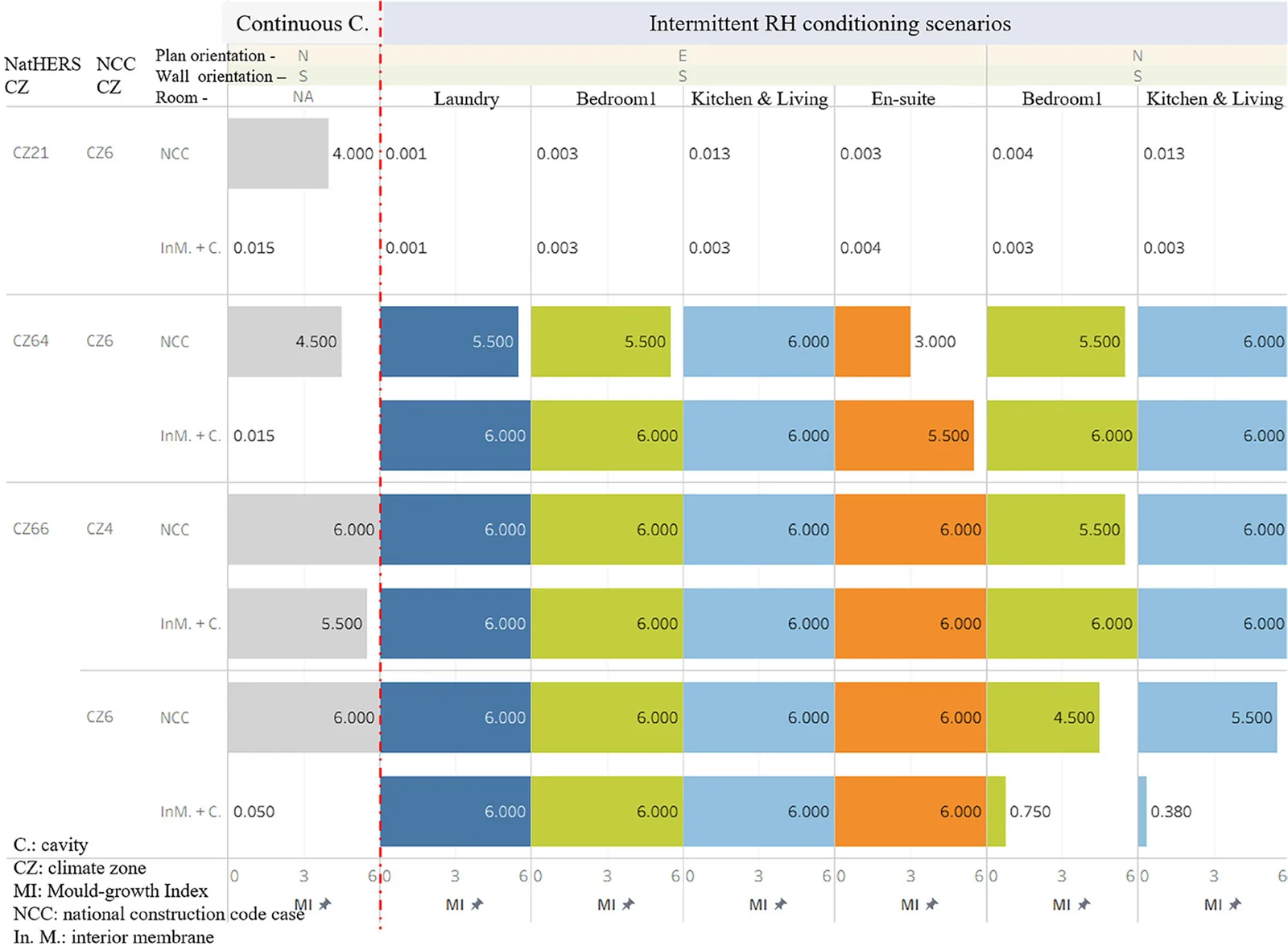
Comparison of bio-hygrothermal results for the brick veneer wall under the two studied conditioning regimes.

Conversely, the results for CZ64 exhibited greater variability than those observed in the preceding CZs. All cases consistently produced higher predicted MI values than their corresponding ASHRAE benchmarks, indicating elevated mould risk across all assemblies, yielding MI values higher than those under continuous conditioning. Notably, the inclusion of the interior vapour control layer was unexpectedly associated with generally higher MI values. This suggests that, under the intermittent conditioning without RH control in CZ64, the application of an interior vapour control layer may adversely affect hygrothermal performance, warranting further investigation into its interaction with orientation and operational conditions.

For CZ66 under NCC-CZ4, MI values increase or remain consistently high with mitigation, reaching 5.5–6.0. Under NCC-CZ6, orientation-dependent behaviour is observed: east-facing assemblies show no change in MI with mitigation, while north-facing assemblies experience a substantial reduction in MI values to very low levels following the application of interior vapour control, within the 3.0 acceptable benchmark. Overall, brick veneer exhibited a higher risk of mould growth than CFCS. In summary, both wall systems showed substantially different performance patterns across the two conditioning regimes.

## Discussion

The results of this study clearly indicate a pronounced difference in moisture and mould risk under intermittent conditioning without RH control, compared with corresponding continuous conditioning scenarios. Collectively, the results suggest that the influence of conditioning patterns and uncontrolled RH on hygrothermal performance has been underestimated, and that a gap exists between current moisture-management approaches prescribed in national and international guidelines and the operating conditions commonly found in residential buildings. Importantly, mitigation strategies previously identified as effective did not consistently achieve the expected performance under intermittent conditioning without RH control. In particular, the interior vapour control layer exhibited highly variable behaviour across CZs, room types, orientations and wall assemblies. In several cases, interventions intended to reduce moisture risk were associated with increased predicted MI values.

The hygrothermal performance between the two conditioning regimes stems from the dynamic shift in vapour pressure gradients across the building envelope during non-operational hours. Under continuous conditioning, a stable indoor thermal environment maintains steady vapour pressure differentials, allowing the assembly to establish a predictable drying path. However, when conditioning is operated intermittently without RH control, the indoor environment floats dynamically. Internal moisture loads, generated by occupant activities such as cooking or respiratory vapour, accumulate within the sealed, highly insulated 7-star envelope. This causes indoor RH to spike. When this high-vapour-pressure indoor air interacts with a dropping outdoor temperature gradient, the vapour pressure drives moisture deep into the wall cavity. Because the thermal energy required to drive drying is absent during off-hours, moisture accumulates progressively within the interstitial microclimate.

These findings challenge the implicit assumption in current regulatory frameworks that continuous conditioning or simplified steady-state metrics can accurately predict moisture risks. Standard international methodologies, such as ASHRAE 160, and local pathways, such as NatHERS, traditionally rely on standardised internal boundaries. However, as demonstrated by previous literature, Australian housing surveys and building authority reports [8,37,38,46,52], the baseline reality of Australian residential occupancy consists of highly intermittent, localised space conditioning. By ignoring these transient operational patterns, current design guidelines create a significant performance gap between regulatory compliance and real-world durability.

This study aligns with emerging international literature that pairs simulation profiles with empirical field-measured data [27,28,31,53–55], which demonstrates that tighter, energy-efficient envelopes are highly sensitive to internal moisture management. While earlier studies conducted under continuous baselines deemed interior vapour membranes universally effective, this research proves that under real-world intermittent schedules, their performance is heavily contingent on microclimatic context [8,18,32].

The findings of this study carry immediate practical implications for the Australian housing sector as it transitions through the 7-star energy efficiency standards, demonstrating that national regulatory frameworks like the NCC must shift from static macroclimatic assumptions towards dynamic assessment protocols that account for real-world intermittent conditioning profiles and room-specific microclimates. For design practitioners and builders, the evidence reveals that high-performance envelopes require zone-, climate-, conditioning- and material-specific solutions rather than a ‘one-size-fits-all’ approach; specifically, caution must be exercised when specifying restrictive interior vapour control layers, as their hygrothermal performance is highly inconsistent under different conditioning regimes and can notably worsen the predicted mould growth risks.

The simulation results consistently demonstrated increased mould growth risk under intermittent conditioning without RH control. While these findings should be interpreted within the context of inherent modelling uncertainties, they are broadly consistent with previous studies that reported similar moisture accumulation trends and elevated mould risk under fluctuating indoor environmental conditions. Existing research has shown that hygrothermal simulation tools can provide reasonable agreement with field measurements when appropriate material properties and boundary conditions are applied [28,31,52]. Several limitations should be acknowledged. The study was limited to Australian climates and occupancy schedules based on Australian regulatory assumptions, which may not fully represent the diversity of real occupant behaviour. The simulations relied on material property databases, mould growth sensitivity parameters and idealised boundary conditions, including the assumption of ‘perfect wall’ construction without air leakage, water ingress, workmanship defects, material degradation or construction failures. Indoor RH was derived from simulated indoor climate conditions and occupancy assumptions; however, in practice, RH varies dynamically with temperature, occupant activities, moisture generation and ventilation effectiveness. Although previous studies have demonstrated reasonable agreement between hygrothermal simulations and field measurements, the absence of monitored occupancy, indoor environmental and building performance data remains a limitation and highlights the need for future validation and calibration using long-term field measurements. Additional limitations include the use of a single reference meteorological year, which does not capture long-term climate variability or extreme events, the exclusion of shower-generated moisture in bathroom simulations due to software constraints and the mould assessment limit of MI = 6, although mould growth may continue under favourable conditions. In addition, due to limitations in AccuRate, moisture generation from showering was not included in bathroom simulations.

Future research should investigate how diverse conditioning patterns influence the efficacy of moisture-management strategies across a broader range of macroclimates, building typologies and occupancy scenarios. Furthermore, long-term empirical field monitoring under intermittent, uncontrolled RH conditions is critical to validate these simulation results and calibrate predictive hygrothermal models for real-world applications. While the latest version of ASHRAE Standard 160 (2016–2021) acknowledges the importance of humidity control, Sections 4.3.2.2 and 4.3.2.3 specify a default indoor RH setpoint of 50% when no humidity-control setting is defined. This value should not be interpreted as a peak RH limit or a cumulative moisture exposure criterion; rather, it functions as a design reference condition for establishing indoor boundary conditions. Furthermore, Section 4.3.2.1.3 requires that the effectiveness of intermittent ventilation systems be considered in accordance with ANSI/ASHRAE Standard 62.2. The findings of the present study suggest that transient indoor humidity conditions arising from intermittent residential operation may have a greater influence on mould risk than is captured by simplified design assumptions. Therefore, more explicit guidance on occupancy-driven conditioning patterns, transient RH behaviour and moisture-risk assessment may be required to support the design of energy-efficient residential buildings. Future moisture-management guidelines should explicitly address intermittent conditioning scenarios and incorporate considerations of occupancy behaviour, indoor RH control and ventilation effectiveness. Ultimately, the design of future energy-efficient housing should consider energy performance and indoor environmental health as equally important and interdependent objectives.

## Conclusion

This study investigated the hygrothermal and bio-hygrothermal performance of typical Australian 7-star NatHERS timber-framed clay masonry veneer and CFCS-clad external wall systems under intermittent conditioning without RH control. Our findings reveal a critical divergence in performance between the realistic, unmanaged moisture profiles common in Australian households and the continuous conditioning regimes prescribed by international standards such as ASHRAE 160 and DA07.

The results demonstrate that intermittent conditioning without RH control introduces unique risks for interstitial moisture accumulation and mould growth, particularly in cold-humid climates (CZ66). Crucially, this research highlights that standard mitigation strategies, specifically the installation of interior vapour control layers, may be counterproductive or even detrimental when subjected to uncontrolled RH and intermittent usage. While wall orientation and room functionality influence moisture dynamics, the primary driver of bio-hygrothermal risk is the synergistic interaction between local climate and specific operational conditioning patterns, further highlighting the need for microclimate- and orientation-informed simulation processes.

These findings expose a fundamental misalignment between current Australian building thermal performance and building conditioning regulations and the verification methods required to assess hygrothermal risks. The national and international drive towards airtight, high-performance envelopes often prioritises energy efficiency at the expense of moisture management. Beyond Australia, these findings serve as a critical alert for international standards: as climate warming increases and energy resources become more constrained, there is a broader shift from continuously conditioned to intermittently conditioned building interiors. In addition, this research highlights the impacts of diverse climatic zones, from coastal to more inland-based locations, with different diurnal conditions for location-based temperature and RH. By neglecting the impact of uncontrolled RH and dynamic occupancy, current guidelines significantly increase the risk of structural degradation and adverse health outcomes, directly undermining the objectives of resilient, net-zero construction.

To achieve true building high performance, the focus must move beyond a singular focus on thermal energy and adopt an integrated moisture-risk framework. There is an urgent need for Australian building regulations, and those in other jurisdictions where intermittent conditioning is the cultural or economic norm, to formally incorporate RH management to account for dynamic occupancy patterns and more specific location-based multi-year climate data. Future regulatory frameworks must prioritise a holistic balance, integrating energy efficiency with robust ventilation and advanced moisture control, to ensure that the transition to sustainable construction does not compromise the health and longevity of the global built environment.

## Data Availability

The datasets generated and/or analysed during the current study are available from the corresponding author on reasonable request.
